# 

**Published:** 1888-07

**Authors:** 


					﻿N O T IC E.
All articles for publication, books for review, business communications, etc.,
must be sent to Editors, 1133 Spruce Street, Philadelphia.
Subscribers will please notice that this Journal will be sent until arrears
are paid and it is ordered discontinued.
** Am I my brffaher’s keeper?” You are to the extent of
being negligent of his best interests if you let him be igno-
rant of the great work THE HOYLCEOPATHIC PHYSI-
CIAN is doing. See that your friends know of this work.
'	.	WANTED.
The following back numbers of The Homceopathic Physician are wanted;
twenty-five cents will be paid for each number received in good condition.
Please look over your odd journals and see if you can help us. Write your
name on the wrapper. Volume eight will be sent In exchange for either
volume one, two, three, or six.
Address The Homceopathic Physician,
1123 Spruce Street, Philadelphia.
Volume First: February, April, and June.
Volume Second: October, November, and December.
Vol time Third : Apri 1.
Volume Sixth :: January.
				

